# Parent, Teacher and Observational Reports of Emotional and Behavioral Problems in Young Autistic Children

**DOI:** 10.1007/s10803-021-05421-x

**Published:** 2022-01-13

**Authors:** Melanie Palmer, Joanne Tarver, Virginia Carter Leno, Juan Paris Perez, Margot Frayne, Vicky Slonims, Andrew Pickles, Stephen Scott, Tony Charman, Emily Simonoff

**Affiliations:** 1 Department of Child and Adolescent Psychiatry, King’s College London, Institute of Psychiatry, Psychology & Neuroscience, London, UK; 2 Department of Psychology, School of Life and Health Sciences, Aston University, Birmingham, UK; 3 Department of Biostatistics and Health Informatics, King’s College London, Institute of Psychiatry, Psychology & Neuroscience, London, UK; 4 Newcomen Neurodevelopmental Centre, Evelina Children’s Hospital, Guy’s and St Thomas NHS Foundation Trust, London, UK; 5 Service for Complex Autism & Associated Neurodevelopmental Disorders, South London and Maudsley NHS Foundation Trust, London, UK; 6 Department of Psychology, King’s College London, Institute of Psychiatry, Psychology & Neuroscience, London, UK

**Keywords:** Autism, Child emotional and behavioral problems, Informants, Agreement, Discrepancies

## Abstract

Emotional and behavioral problems (EBPs) frequently occur in young autistic children. Discrepancies between parents and other informants are common but can lead to uncertainty in formulation, diagnosis and care planning. This study aimed to explore child and informant characteristics are associated with reported child EBPs across settings. Participants were 83 4–8-year-old autistic children and their parents and teachers in the Autism Spectrum Treatment and Resilience (ASTAR) study. Questionnaires of child EBPs were completed by parents and teachers, and self-reported parenting stress and wellbeing measures were obtained. An observation of parent–child/researcher-child interaction was also completed. Parents reported more EBPs than teachers and parent-teacher agreement was low, particularly for emotional problems. Greater parenting stress and being verbal was associated with more parent- but not teacher-reported EBPs. More observed behaviors that challenge were displayed by minimally verbal children. More parenting stress could be associated with the presence of more EBPs in the home; alternatively, parenting stress may confound reports. It is essential for assessments of EBPs in autistic children to take a multi-informant approach. Better understanding of the associations between informant characteristics and informant discrepancies of EBPs is needed.

**Supplementary Information:**

The online version contains supplementary material available at 10.1007/s10803-021-05421-x.

## Introduction

Autism Spectrum Disorder is a neurodevelopmental condition associated with difficulties in social communication, and the presence of restricted and repetitive interests and sensory processing difficulties (American Psychiatric Association, 2013). Emotional and behavioral problems (EBPs) are common in autistic^1^ individuals (Lai et al., 2019). As many as 70% of autistic children may meet criteria for a co-occurring psychiatric condition; attention deficit hyperactivity disorder (ADHD), anxiety disorders and oppositional defiant disorder (ODD) are the most prevalent (Salazar et al., 2015; Simonoff et al., 2008). EBPs can also be associated with behaviors that challenge (BTC).^2^ For example, anxiety is associated with oppositional behavior, hyperactivity, aggression and meltdowns (Sukhodolsky et al., 2020; Tarver et al., 2021a). For autistic individuals EBPs persist over time (Simonoff et al., 2013; Stringer et al., 2020), impact on quality of life (Mason et al., 2018) and are associated with more parental distress (Yorke et al., 2018).

### Informant Discrepancies in the Assessment of EBPs

To conduct a thorough assessment of EBPs in childhood, guidelines suggest assessment of impairment across settings (National Institute for Health & Care Excellence, 2013). The use of multiple informants (e.g. parent and teacher) and naturalistic observation ensures a comprehensive understanding of the individual across contexts (Mash & Hunsley, 2005). Informant discrepancies, where informants provide different reports of similar constructs, are expected and commonplace during assessments of EBPs in autism (Stratis & Lecavalier, 2015). This can lead to uncertainty in clinical decision making and treatment planning, but may give information on factors driving or maintaining EBPs.

There are a range of factors, not related to measurement error, that may influence reporting of child EBPs and lead to informant discrepancies. A key factor is situational specificity (De Los Reyes et al., 2013); the nature of the settings in which different individuals interact with the child could impact how informants understand and report EBPs. For example, the structured nature of the school environment may be associated with different displays of EBPs at school. Discrepancies across reports may therefore yield important information on how the child behaves in different contexts and can be used to inform environmental modification and intervention for EBPs.

Levels of EBPs may vary across settings and literature comparing reports from parents and teachers of EBPs among autistic children are mixed. Some studies report higher levels of teacher-reported oppositionality and anxiety (Reed & Osborne, 2013), whereas others report higher levels of parent-reported EBPs compared to teachers (Jepsen et al., 2012; Llanes et al., 2020). Although comparable to agreement in the general population (Achenbach et al., 1987), levels of agreement between parents and teachers on reports of EBPs for autistic children are often moderate at best (Stratis & Lecavalier, 2015). Whilst it is likely that situational specificity is playing a role, parent-teacher agreement in autism remains low when teachers and parents are observing and reporting on child behavior in the home (Reed & Osborne, 2013). This indicates that factors other than situational specificity are likely to influence reports of EBPs and better understanding of these factors is warranted. Furthermore, few studies have explored how observational measures of child behavior, often considered the ‘gold standard’, are associated with other informant reports of EBPs among autistic children.

### Child Characteristics Associated with Informant Reports

#### IQ and Verbal Language Level

Verbal language level varies greatly in autism, and IQ, which shows strong overlap with verbal language level (Bal et al., 2016), is associated with reports of EBPs in autism. Parents of minimally verbal autistic children report higher levels of child irritability and hyperactivity than parents of verbally fluent children (Fok & Bal, 2019). However, inconsistent relationships between informant reports of levels of anxiety and IQ have been found (see Kent & Simonoff, 2017; Mingins et al., 2020 for reviews); some studies report greater parent-reported anxiety in autistic individuals with higher IQ (Chandler et al., 2016; Hallett et al., 2013; Salazar et al., 2015) and others do not find this association (Simonoff et al., 2008). Assessment of emotional problems is likely to be more difficult in minimally verbal individuals due to limited ability to communicate internal states. However, when considering the effects of IQ on informant agreement, evidence suggests that as IQ increases, informant agreement about autistic children’s emotional problems decreases (Stratis & Lecavalier, 2015). It was suggested that individuals with lower IQ show less varied behavior across contexts, or that caregiver discussions about EBPs may be more frequent in children and young people with low IQ leading to greater agreement.

#### Autism Severity

There are also mixed findings as to whether autism severity is associated with levels of reported EBPs. Some evidence suggests autism severity is associated with increased risk of meeting diagnostic criteria for agoraphobia and night terrors on a parent-informed psychiatric interview, but not other common co-occurring conditions including social anxiety and disruptive behavior disorders (Salazar et al., 2015). In another population-based cohort of autistic children, autism severity was not associated with increased risk for co-occurring conditions (Simonoff et al., 2012). Furthermore, Stratis and Lecavalier (2017) found no evidence that autism severity was associated with parent-teacher agreement of child EBPs; however, Levinson et al. (2021) found that autism severity predicted disagreement between parent and teacher reports of EBPs.

#### Severity of EBPs

Informant discrepancies could be a marker of the severity of child EBPs. Discrepancies may be more likely if child EBPs are less severe and/or frequent leading to differing interpretations between informants (De Los Reyes et al., 2009). However, in non-autistic children, there is evidence that occurrences of observed behavior specific to one context and interaction partner (e.g. parent or examiner) are related to higher levels of parent- or teacher-reported disruptive behavior (De Los Reyes et al., 2009). To the best of our knowledge, relationships between observations of behavior in a third context and parent and teacher reports of EBPs has not been explored in autism. This provides an important area for autism research given that children less able to modulate behavior across multiple contexts may be more likely to show more stable and severe EBPs (Granic & Patterson, 2006; Jones et al., 1975).

### Informant Characteristics Associated with Informant Reports

#### Parenting Stress and Wellbeing

Reports of EBPs are also likely to be influenced by a host of characteristics related to the informant, such as stress and wellbeing. Parents of autistic children report higher levels of stress than parents of non-autistic children and other developmental disabilities (Estes et al., 2013) and there is an established bidirectional relationship between parental distress and child EBPs in autism (Yorke et al., 2018). Parenting stress and wellbeing might be associated with reports of EBPs in several ways, leading to disagreement between different informants. First, parenting stress may reduce parental capacity to cope, perhaps biasing parental views of child behavior (Najman et al., 2000). Second, children of distressed parents may show elevated levels of problem behavior that is restricted to the home environment or during interactions with parents. Third, the relationship between parental distress and parent-reported child EBPs may be due to shared method variance (Podsakoff et al., 2003). There is some evidence to show that levels of parenting stress are associated with parental reports of certain domains of child functioning, such as behavioral problems, but not linked with clinician ratings of child functioning (Schwartzman et al., 2021). Exploration of relationships between parenting stress and reports of EBPs in other settings (such as teacher-reported EBPs and observational measures of child behavior) is limited. If parenting stress is associated with reports of EBPs by other informants, this may suggest that stress does not play a role in confounding parental reports of EBPs, or that parenting stress is elevated because EBPs are primarily displayed at home.

#### Teacher Characteristics Associated with Informant Reports

Various factors can also influence teacher reports of child EBPs among autistic children and could result in different reports of EBPs when compared to parents. Teacher reports are likely to be influenced by the structure and expectations of classroom activities and situations in schools. Further, teachers observe children in situations that parents may be less likely to see, including interactions with peers and performance in directed tasks. Another factor may be the type of school a teacher is employed in, and research has found that teachers working with autistic children in mainstream settings report higher levels of general, but not social anxiety, compared to teachers in specialist education settings (Adams et al., 2018). It may be that EBPs may be more commonly observed, and therefore less salient to teachers in specialist educational settings; saliency is a factor found to impact reported EBPs (Karver, 2006). Differences could also reflect a commonly reported, albeit inconsistent, finding of a relationship between higher IQ and greater anxiety in autism (e.g. Chandler et al., 2016), or true variations in anxiety displayed across contexts.

### Aims of the Current Study

Given the varied findings in the literature of factors associated with reports of EBPs in autistic children, there were three key objectives of this study. First, we compared levels of EBPs in autistic children reported by parents and teachers. We hypothesized that parents would report higher levels of EBPs than teachers. Second, we explored whether child and informant characteristics were associated with levels of parent-, teacher- and researcher-rated observed EBPs. We hypothesized that less verbal language, greater autism severity and higher rates of researcher-rated observed behaviors that challenge (BTC; e.g. non-compliance, hyperactivity) would be associated with more parent- and teacher-reported EBPs, and that autism severity and less verbal language would be associated with more researcher-rated observed BTC. We expected that higher self-reported parenting stress and lower parental wellbeing would correlate with greater parent-reported EBPs, and that special school placement would be associated with lower teacher-reported EBPs as teachers in these settings are likely to have differing thresholds and have more support for child EBPs. We did not have a specific hypothesis for whether parent characteristics would be associated with teacher-reported and researcher-rated observed child EBPs, but instead explored the associations between parental reports of parenting stress and wellbeing and these measures. Finally, we explored agreement between parent and teacher reports of EBPs and whether informant agreement between teachers and parents varied according to score severity. We hypothesized that parent-teacher agreement would be modest, but that agreement would increase as the severity of parent and teacher rated EBPs increased.

## Method

### Participants

Participants were 83 young autistic children and their parents (see Table 1 for further details) participating in the Autism Spectrum Treatment and Resilience (ASTAR) study (Charman et al., 2021; Palmer et al., 2019), as part of the Improving Autism Mental Health (IAMHealth) research program (https://iamhealthkcl.net). Most children were male (*n* = 71, 85.5%) and the mean age was 6.70 years (*SD* = 1.21 years). Verbal language levels varied across the sample; 39 (47.0%) were minimally verbal and 44 (53.0%) were verbal (see description of verbal ability grouping in measures section below). In addition, 76 education professionals (mainly Class Teachers, but occasionally a school Special Educational Needs Coordinator, Learning Support Assistant, or Head Teacher; referred to hereafter as ‘teachers’ for simplicity) completed questionnaires about the children.

**Table 1:** Characteristics of the sample (*N* = 83)

Demographic characteristics	*n*	%
Child gender		
Male	71	85.5%
Child ethnicity^a^		
White	44	53.7%
Asian/Asian British	8	9.8%
Black/Asian British	14	17.1%
Mixed/Multiple ethnicities	15	18.3%
School placement^b^		
Specialist school	32	39.0%
Mainstream school	37	45.1%
Specialist unit in mainstream school	13	15.9%
Parent informant^c^		
Mother	76	91.6%
Father	5	6.0%
Grandmother	2	2.4%
Parental education level^c^		
No formal qualifications	10	12.2%
General Certificate of Secondary Education or equivalent	9	11.0%
General Certificate of Education Advanced Level (A levels) or equivalent	10	12.2%
Vocational qualifications (NVQ, City and Guilds or equivalent)	13	15.9%
Undergraduate tertiary degree	16	19.5%
Postgraduate tertiary degree	24	29.3%
Parental employment status^b,c^		
Not in paid employment	40	48.8%
In part-time paid employment	25	30.5%
In full-time paid employment	17	20.7%
Annual household income^d^		
Less than £20,000	23	35.9%
£20,000–£39,999	14	21.9%
£40,000–£59,999	8	12.5%
£60,000–£79,999	10	15.6%
Greater than £80,000	9	14.1%

Other clinical characteristics	*M*	*SD*	Observed range
Observed child behaviors that challenge rate/ per minute (OSCA–ABP)	2.00	1.81	0.05–8.74
Autism severity (ADOS–2 CSS)	7.53	1.90	1–10
Adaptive behavior (ABAS–3)	62.51	12.79	45–100
Parenting stress (APSI)^b^	22.46	9.58	5–49
Parental wellbeing (SWEMWBS)^b^	21.08	4.20	7–30.70

*N* = 83, valid % reported

*ABAS–3* Adaptive Behavior Assessment System – 3rd edition, *ADOS–2 CSS* Autism Diagnostic Observation Schedule, second edition, Calibrated Severity Score (0–10), *APSI* Autism Parenting Stress Index (0–65), *OSCA–ABP* Observation Schedule for Children with Autism–Anxiety, Behaviour and Parenting (0 +), *SWEMWBS* Short Warwick-Edinburgh Mental Well-being Scale (7–35)

^*a*^*n* = 81 Data was missing for one child and one did not wish to answer. White = English/Welsh/Scottish/Northern Irish/Irish/British/Other White ethnicity, Black/Black British = African/Caribbean/Other Black ethnicity, Asian/Asian British = Indian/Pakistani/Bangladeshi/Chinese/Other Asian ethnicity, Mixed/Multiple ethnicities = White and Black Caribbean/White and Black African/White and Asian/Other Mixed ethnicity

^b^*n* = 82. Data was missing for one child

^c^Refers to parent involved in completing the questionnaires/observation

^d^*n* = 64. Data was missing for one child and 18 did not wish to answer

### Procedure

Participants were enrolled in the ASTAR study, a non-randomized feasibility study followed by a pilot randomized controlled trial (RCT) of two novel parent-mediated group interventions. Ethical approval for the study was granted from NHS Camden and Kings Cross Research Ethics Committee (ref: 16/LO/1769). Written informed consent was obtained from all participating parents and teachers and child assent was obtained wherever appropriate.

Twenty-one parent–child dyads participated in the feasibility study and 62 in the pilot RCT, recruited from local autism diagnostic teams, education professionals, support groups, consented databases, and self-referral routes in four boroughs of South London. To be eligible to take part, the child had to have a clinical diagnosis of an Autism Spectrum Disorder and be between 4 years 0 months and 8 years 11 months at randomization. The study was designed to be as inclusive as possible, so no exclusion based on levels of child EBPs was used; nor for verbal language level or IQ. Additional inclusion/exclusion criteria can be found in Palmer et al. (2019).

Data collected during the baseline assessments were used in the current study. Parents completed questionnaires on their child’s EBPs and their own parenting stress and wellbeing. With parental consent, the child’s teacher completed questionnaires about the child’s EBPs at school. The baseline assessment also involved a research visit where observations of parent–child/researcher-child interaction and autism severity were obtained.

### Measures

#### Sample Characterization

Demographic information about the family was obtained using a bespoke questionnaire. Autism severity was measured using the Autism Diagnostic Observation Schedule – 2nd edition (ADOS–2, Lord et al., 2012). Verbal language grouping was based on the ADOS–2 module completed with the child (minimally verbal = Module 1 vs. verbal = Module 2 or 3). To measure adaptive skills and functioning, the Adaptive Behavior Assessment System – 3^rd^ edition (ABAS–3, Harrison & Oakland, 2015) was completed by parents. Scores on the ADOS–2 and the ABAS–3 for the sample are presented in Table 1.

#### Parent-Reported Child Emotional and Behavioral Problems

Parent-reported child irritability and hyperactivity was measured using the Irritability and Hyperactivity subscales of the Aberrant Behavior Checklist (ABC, Aman & Singh, 1994). The ABC is an informant report measure of EBPs developed for use in populations with developmental disabilities and is widely used in autism intervention studies (e.g. Bearss et al., 2015). Items were rated on a 4-point scale ranging from ‘*not at all a problem*’ to ‘*the problem is severe in degree*’ and summed to produce total subscale scores, with higher totals signifying more irritability and hyperactivity. The ABC has established reliability and validity (Aman & Singh, 1994).

Parent-reported child EBPs were also measured using the Emotional (19 items; e.g. spends a lot of the day feeling worried) and Behavioral (15 items; e.g. too much energy) Problems subscales of the Assessment of Concerning Behaviour (ACB). The ACB is a new measure developed specifically for use in autistic children and young people (Tarver et al., 2021b). Items were rated on a 5-point sliding scale anchored by opposing responses (‘*not at all*’ to ‘*very much*’) and summed to produce total subscale scores. Higher scores indicate more EBPs. Good internal validity for parent-reported ACB Emotional (*α* = 0.87) and Behavioral Problems (*α* = 0.86) subscales was found for this sample.

#### Teacher-Reported Child Emotional and Behavioral Problems

Teachers completed the ABC Irritability and Hyperactivity subscales (Aman & Singh, 1994) and the teacher version of the ACB (Tarver et al., 2021b). In this sample, internal validity of teacher ACB Emotional and Behavioral Problems was *α* = 0.77 and *α* = 0.88 respectively.

#### Observed Behaviors that Challenge

The rate of child behaviors that challenge (BTC) observed during an 18 to 22-min structured parent–child/researcher–child interaction, the Observation Schedule for Children with Autism Spectrum Disorders – Anxiety, Behaviour and Parenting (OSCA–ABP, Palmer, Paris Perez et al., 2020), were coded from video-recordings by researchers. During the observation, six parent-led (shared task, shared game, separation, reunification and tidy up, homework sheet and walking along a line) and two researcher-led (mystery box and an unopenable snack jar with reward delay) tasks were completed that aimed to elicit observable BTC by tapping into uncertainty and novelty, transition, turn taking, sensory processing, compliance, frustration and reward delay, see Palmer, Paris Perez et al. (2020) for further details. The specific materials used in each task were differentiated by child verbal ability group based on the ADOS–2 assessment modules, to take into account differences in expressive language and age, but were designed to be analogous in function. The frequencies of a range of child behaviors (destructive behavior, aggression towards themselves and others, frustrated vocalizations, non-compliance, avoidance and reassurance seeking) observed during the OSCA–ABP were coded and summed to produce the total child BTC score. As the duration of the measure varied, the rate of child BTC per minute was calculated by dividing the total BTC count by the duration. The OSCA–ABP child BTC rate has demonstrated good inter-rater reliability amongst the current sample of verbal (*ICC* = 0.92, 95% *CI* = 0.88, 0.96) and minimally verbal children (*ICC* = 0.77, 95% *CI* = 0.66, 0.89) (Palmer, Paris Perez et al., 2020), and provides a global measure of EBPs, as it covers both anxious and disruptive behavior.

#### Parenting Stress

Parenting stress associated with core and co-occurring symptoms of autism was measured using the Autism Parenting Stress Index (APSI, Silva & Schalock, 2012). Thirteen items were rated on a 5-point scale (‘*not stressful*’ to ‘*so stressful we feel we can’t cope*’), summed to produce a total score with higher scores indicating more parenting stress. The APSI has acceptable internal validity and test–retest reliability (Silva & Schalock, 2012).

#### Parental Wellbeing

Parents reported on their own wellbeing using the Short Warwick Edinburgh Mental Well-Being Scale (SWEMWBS, NHS Health Scotland, University of Warwick and University of Edinburgh, 2008). The SWEMWBS consists of seven positively worded items that tap into wellbeing, summed to produce a total score with higher scores indicating higher wellbeing. The SWEMWBS construct and convergent validity of the measure has been established (Ng Fat et al., 2017).

### Data Analysis

Data analysis was conducted in Stata 15 (StataCorp, 2015). All variables were assessed for normality. Teacher ABC child irritability scores violated the assumptions of normality and where possible, the appropriate non-parametric tests were used. Where there was no non-parametric alternative, we conducted sensitivity analysis using the log transformation of the variable. The interpretation of the results was unchanged when using the log-transformed variable, so the untransformed variable was retained and used for all parametric analyses. First, we examined differences in mean parent- and teacher-reported scores on the ABC and ACB subscales using paired sample *t*-tests and Cohen’s *d* was used to calculate the size of the effects between the groups.

Next, we used a hypothesis-driven approach to test whether child and informant characteristics were associated with levels of parent-, teacher- and researcher-rated observed EBPs. First, we explored the correlations between all potential factors (i.e. child and informant characteristics) and measures of EBPs. To dichotomize school placement, specialist units in a mainstream school were included with mainstream schools, and specialist only schools were separate. This is consistent with previous research that has combined special units in mainstream schools in this way (e.g. Simonoff et al., 2019).

We then conducted a series of multivariate multiple linear regression models using the *sem* command to explore the strength of association between each factor and both parent and teacher-reported EBPs whilst controlling for the other characteristics. As parent- and teacher-reports were obtained using the same measure, they could be entered into the same model (one for each subscale), which increased efficiency and reduced type 1 errors from multiple testing. Where significant associations were found between an independent and dependent variable (either parent- or teacher-reported scores), post-estimation tests were used to compare the coefficients (i.e. testing if the strength of the association between an independent variable and parent-reported scores was significantly different from the association between that same variable and teacher-reported scores).

As observed child BTC were rated on a different scale to the measures completed by parents and teachers, associations with observed BTC were tested using a univariate multiple linear regression model. Independent variables included child verbal language, parenting stress and parental wellbeing. School placement was not included in this regression given the overlap between verbal language grouping and school placement and we did not have a clear hypothesis about how school placement would influence researcher-rated observed BTC above and beyond verbal language.

Inter-rater agreement between parents and teachers was explored in two ways. First, we obtained the intraclass correlation coefficients (*ICC*s) and 95% confidence intervals (*CI*s) to assess agreement across parent and teacher scores on the ABC and the ACB. *F* tests were used to test whether the ICC were significantly different from zero. Bland–Altman plots (BA, Bland & Altman, 1995) were also produced to visually depict systematic trends in informant agreement across the range of scores on the ABC and ACB subscales. We tested whether parent and teacher agreement was associated with score severity on each subscale using Spearman’s rho (as in Bland & Altman, 1995).

## Results

### Group Differences Between Parent- and Teacher-Reported Emotional and Behavioral Problems

Table 2 displays the descriptive statistics and the results of the *t*-tests comparing parent and teacher scores on the ABC and ACB subscales and associated effect sizes. As hypothesized, parents reported significantly more irritability and hyperactivity on the ABC and more emotional and behavioral problems on the ACB compared to teachers; all effect sizes were moderate.

**Table 2:** Differences in mean scores for parent- and teacher-reported child emotional and behavioral problems and inter-rater agreement across measures

Measure	Descriptives			Differences in parent-teacher means and inter-rater agreement								
	Parent-reported child emotional and behavioral problems^a^			Teacher-reported child emotional and behavioral problems^b^								
	*M*	*SD*	Observed range	*M*	*SD*	Observed range	Tests comparing mean parent and teacher reports^b^			Intraclass correlation coefficients (*ICC*)^b^		
							*t* or *W*	*p*	*d*	*ICC*	95% *CI*	*p*
Irritability (ABC)	16.37	10.30	0–44	9.16	10.08	0–44	4.09^c^	< .001	0.50	.16	–.04, .36	.047
Hyperactivity (ABC)	24.09	13.34	1–48	16.93	12.51	0–47	3.89	< .001	0.45	.30	.08, .49	.001
Behavioral problems (ACB)	22.65	11.43	2–49	16.68	11.21	0–56	3.67	< .001	0.42	.27	.06, .47	.003
Emotional problems (ACB)	21.48	13.09	0–59.11	14.66	8.92	1–42	3.76	< .001	0.43	.04	–.15, .24	.350

*N* = 83. ABC = Aberrant Behavior Checklist (Irritably 0–45; Hyperactivity 0–48); ACB = Assessment of Concerning Behaviour (Externalizing 0–60; Internalizing 0.76)

^a^*n* = 82. Data was missing for one child

^b^*n* = 76. Teacher data was missing for seven children

^c^Non-parametric alternative to paired *t*-test (Wilcoxon signed-rank test) and Pearson’s correlation (Spearman’s rho) used due to the teacher ABC irritability variable being positively skewed. Possible range of scores for each measure are in brackets below

### Factors Associated with Parent- and Teacher-Reported Emotional and Behavioral Problems

Supplementary Table S1 displays the bivariate correlations between the child and informant characteristics and parent-, teacher- and researcher-reports of child EBPs (see Table 1 for descriptives of the clinical measures). Parenting stress and wellbeing were significantly positively correlated with the majority of parent-reported EBPs (*r*’s ranged from 0.37–0.61, *p*’s all < 0.001 and *r*’s were − 0.20– − 0.51, *p*’s from 0.071 to < 0.001 for parenting stress and wellbeing, respectively). Observed BTC was positively correlated with teacher-reported behavioral problems on the ACB (*r* = 0.23, *p* = 0.048).

**Fig. 1:**
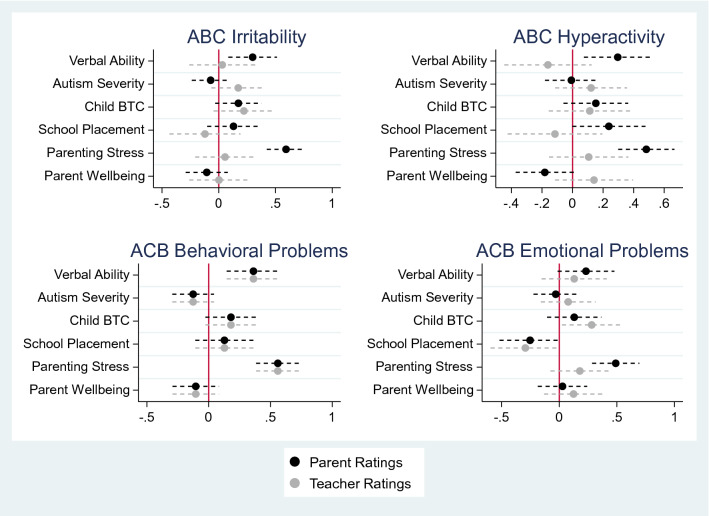
Forest plots showing the standardized coefficients for the multivariate regression models predicting parent- and teacher-reported EBPs

Table 3 displays the standardized coefficients and their associated *p* values for the multivariate linear multiple regression models examining parent- and teacher-reported EBPs. Figure 1 is a forest plot showing the standardized coefficients for these models.

**Table 3:** Regression analyses predicting parent- and teacher-reported child emotional and behavioral problems and researcher-rated observed behaviors that challenge

	Outcome																	
	Irritability (ABC)				Hyperactivity (ABC)				Behavioral problems (ACB)				Emotional problems (ACB)				Observed BTC rate (OSCA–ABP)	
	Parent		Teacher		Parent		Teacher		Parent		Teacher		Parent		Teacher		Observed, researcher-rated	
	*Std coeff*	*p*	*Std coeff*	*p*	*Std coeff*	*p*	*Std coeff*	*p*	*Std coeff*	*p*	*Std coeff*	*p*	*Std coeff*	*p*	*Std coeff*	*p*	*Std coeff*	*p*
Verbal language group^a^ (Minimally verbal vs. verbal)	**0.30**	**.006**	0.03	.835	**0.30**	**.009**	–0.16	.269	**0.36**	**.001**	–0.08	.600	0.23	.065	0.13	.368	**-0.50**	** < .001**
Autism severity (ADOS–2 CSS)	–0.07	.400	0.17	.156	–0.01	.930	0.12	.311	–0.13	.144	0.06	.631	–0.03	.757	0.08	.521	0.13	.180
Observed BTC rate (OSCA–ABP)	0.19	.097	0.22	.104	0.15	.158	0.11	.409	0.18	.088	0.22	.101	0.13	.279	**0.28**	**.033**	–	–
School placement (Specialist vs. mainstream)^b^	0.13	.272	–0.12	.446	0.24	.052	–0.11	.468	0.13	.288	–0.09	.592	–0.25	.062	–0.29	.056	**–**	**–**
Parenting stress (APSI)	**0.59**	** < .001**	–0.06	.680	**0.48**	** < .001**	0.11	.424	**0.56**	** < .000**	0.16	.232	**0.49**	** < .000**	0.18	.169	–0.11	.331
Parental wellbeing (SWEMWBS)	–0.11	.263	0.16	.886	–0.18	.063	0.14	.282	–0.10	.276	0.01	.938	0.03	.794	0.12	.341	–0.04	.724

*N* = 76 for parent-teacher comparisons and 82 OSCA–ABP as parent report data was missing for one child. Significant correlations are in bold

*ABC* Aberrant Behavior Checklist, *ACB* Assessment of Concerning Behaviour, *ADOS–2* Autism Diagnostic Observation Schedule, second edition, *APSI* Autism Parenting Stress Index, *OSCA–ABP* Observation Schedule for Children with Autism–Anxiety, Behaviour and Parenting, *SWEMWBS* Short Warwick-Edinburgh Mental Well-being Scale

^a^0 = Minimally verbal; 1 = Verbal

School placement was not included in this model due to substantial overlap in group membership between school placement and verbal ability and we did not hypothesize school placement would influence researcher-rated observation reports. 0 = Special schools; 1 = Mainstream (Mainstream schools + Special units in mainstream schools

Higher parental ABC irritability scores were uniquely associated with the child being verbal and greater parenting stress. Post-estimation tests showed that children whose parents reported greater parenting stress had significantly higher parent-, but not teacher-reported, child irritability (*p* < 0.001). The post-estimation test for verbal language grouping was not significant (*p* = 0.093).

For parent-reported ABC hyperactivity, the child being verbal and greater parenting stress were associated with higher hyperactivity scores. Post-estimation tests showed that these factors were significantly associated with parent (*p* = 0.001 and 0.002 respectively) but not teacher reports of hyperactivity.

For ACB behavioral problems, children who were verbal and had parents reporting greater parenting stress had significantly higher parent-reported behavioral problems; post-estimation tests showed these variables were significant correlates of parent-reported behavioral problems when compared to teacher reports (*p* = 0.004 and *p* = 0.004).

For the ACB emotional problems scores, higher parenting stress was associated with more child emotional problems, with post-estimation tests showing that higher levels of parenting stress were associated with higher levels of parent-reported, but not teacher-reported, child emotional problems (*p* = 0.013). For teachers, more observed BTC was significantly associated with higher teacher reports of child emotional problems, however, post-estimation tests showed that when compared to parent-report, observed child BTC was not significantly associated with teacher-reported emotional problems (*p* = 0.703).

### Factors Associated with Observed Child Behaviors that Challenge

Supplementary Table S1 displays the correlations between the child and informant characteristics and researcher-rated observed child BTC rates. Being minimally verbal was associated with more observable child BTC. The standardized coefficients and associated *p* values for the univariate multiple linear regression model examining researcher-rated observed child BTC rates are also displayed in Table 3. When controlling for other variables, being minimally verbal was the only factor associated with more observable child BTC.

### Agreement between Parent- and Teacher-Reported Emotional and Behavioral Problems

Table 2 presents the *ICC*s between parent and teacher scores and associated *p* values on the ABC and ACB measures. The *ICC*s examining agreement between parents and teachers ranged from 0.04 (child emotional problems) to 0.30 (child hyperactivity). *ICC*s were statistically significant for all variables with the exception of ACB child emotional problems.

Informant agreement and variation in agreement by score severity was further examined using Bland–Altman plots and the accompanying regression analysis (see Supplementary Fig. S1). Supplementary Table S2 presents the mean differences in parent and teacher scores and combined parent-teacher scores. No significant relationships between the difference scores and the average combined scores were found for the ABC irritability, ABC hyperactivity and ACB behavioral problems (see Supplementary Fig. S1 for *ρ* and *p* values). However, for the ACB emotional problems, there was a significant relationship indicating that agreement varied based on severity of emotional problems (*ρ* = 0.29, *p* = 0.011), with a general pattern of larger discrepancy between informants as emotional problem scores increase.

## Discussion

This study compared levels and investigated child and informant characteristics associated with child EBPs and reports from three different informants (parents, teachers and researchers). It also explored agreement between parent- and teacher-reported EBPs.

### Levels of Parent- and Teacher-Reported Emotional and Behavioral Problems

As hypothesized, in the current sample of 4–8-year-old autistic children, parents reported more EBPs than teachers and this pattern held across both emotional and behavioral problems. This aligns with previous literature in non-autistic (Rescorla et al., 2014) and autistic populations (Chandler et al., 2016; Jepsen et al., 2012). The differences in levels of EBPs reported in home and educational settings lends support for the notion of situational specificity, although because the reports are provided by different individuals, other informant characteristics cannot be ruled out. The nature of these different environments is likely to influence which behaviors are displayed. One possibility is that the structured, routine-based school environment reduces the likelihood of a child encountering unexpected events or stimuli, a key trigger for EBPs in autism (Bearss et al., 2015). A further explanation, often described anecdotally by parents and clinicians, is a delayed display of the pressures faced by autistic children during a typical school day. It may be that autistic children are ‘holding in’ manifestations of EBPs (Bearss et al., 2015) in school environments resulting in reports of less EBPs by teachers and greater manifestations of EBPs when with parents. This may only be relevant for a sub-group of autistic individuals who have the cognitive ability to reflect and greater self-control to modify their behavior. Indeed, in this study, the child being verbal (suggesting higher IQ and more cognitive ability) was generally associated with higher parent-, but not teacher-, reported EBPs.

### Factors Associated with Parent- and Teacher-Reported Emotional and Behavioral Problems

We also explored child and informant characteristics associated with parent-, teacher- and researcher-rated EBPs. For emotional problems, higher levels of parenting stress were associated with more parent-reported child emotional problems. For behavioral problems, higher levels of parenting stress and the child being verbal were associated with increased parent-reported behavioral problems. This finding is at odds with previous research that has reported more irritability and hyperactivity among minimally verbal children, albeit in a sample of older autistic children and young people aged 6–18 years (Fok & Bal, 2019). This inconsistent finding could also be due to difficulties in measuring EBPs in autistic children, particularly among those who are minimally verbal or have an intellectual disability (Flynn et al., 2017). However, the relationship between the child being verbal and elevated parent-reported behavioral problems was consistent across the measures used in this study, including the ACB, a measure of EBPs developed specifically for autistic populations and shown to be reliable and valid across the spectrum of developmental ability in autism (Tarver et al., 2021b). Interestingly, parental wellbeing was not a correlate of EBPs. It may be that stress related to the parenting role has a stronger relationship with child EBPs than more general parent wellbeing (e.g. feeling relaxed and confident, can deal with day-to-day problems). This could be especially true given that the parenting stress measure used in this study was specific to stress related to autistic traits and co-occurring EBPs.

That parenting stress is associated with parent-reported EBPs but not teacher nor observed child difficulties in our study is important. This adds to previous research with both non-autistic and autistic children which found parenting stress is associated with greater discrepancies in reports of EBPs between parents and teachers (Langberg et al., 2010; van der Oord et al., 2006). The findings from this study could suggest that stress is influencing parent reports of EBPs in this sample (Najman et al., 2000); that the association between elevated parenting stress and parent-reported EBPs was consistent across multiple parent-reported measures acts as further support for this. However, it is also possible that parenting stress is elevated because EBPs are predominantly challenging in the home context, or in situations when children are more likely to be with their parents (e.g. in the supermarket, when on holiday). If so, the findings of this study may be suggestive of situational specificity, and that young autistic children are more likely to manifest EBPs with their parents. This is not to say that autistic children do not display EBPs at school, but instead, they become more visible when children get home (Bearss et al., 2015). When we tested whether the same characteristics were also associated with teacher reports of EBPs, a different pattern of results was found. None of the child or informant characteristics included in the models were associated with teacher reports of child irritability, hyperactivity, behavioral or emotional problems. Contrary to our hypothesis, verbal ability was not associated with teacher-reported EBPs. On the other hand, being minimally verbal was associated with more researcher-rated observed BTC, which is in line with other literature (e.g. Einfeld et al., 2011). No parental characteristics were associated with the rate of child BTC seen during this observation.

### Agreement Between Parent- and Teacher-Reported Emotional and Behavioral Problems

In general, agreement between parent and teacher reports of EBPs was low. In line with non-autistic populations (e.g. Rescorla et al., 2014), agreement between parents and teachers appeared to be stronger for more observable behaviors (hyperactivity; behavioral problems), with lower agreement found for measures of irritability and emotional problems (domains which include questions interrogating internal states and mood). This pattern of findings is also similar to the levels of agreement reported between parents and teachers of young autistic individuals (Llanes et al., 2020; Stratis & Lecavalier, 2015), although agreement between parents and teachers in the current sample is lower for emotional problems in particular. This may be in part due to the small sample size and lack of precision as confidence intervals were wide.

Agreement was also explored using Bland–Altman plots to visually depict trends in informant agreement. For the measures of child irritability, hyperactivity and behavior problems, there was no relationship between parent and teacher agreement and the severity of EBPs. However, for emotional problems, agreement varied based on how severe the scores were with a general pattern of larger differences between informants as emotional problems increased. This suggests that when emotional problems are reported as problematic in one context, they may not necessarily be problematic in another context. For example, anxiety could be more ‘situation specific’ than hyperactivity among autistic children. Another explanation may be that anxiety presents in atypical ways in autism (e.g. as repetitive or challenging behavior, Bearss et al., 2015; Kerns et al., 2014; Tarver et al., 2021a) and reliance on interpretation may explain lower agreement. However, it is of note that we did not find any association between parent-reported emotional problems and teacher-reported behavioral problems, and vice versa. This suggests emotional problems rated by one informant are not interpreted as behavioral problems by the other.

### Clinical Implications

Parent-teacher agreement on measures of EBPs is modest; young autistic children may show variations in EBPs across settings. A comprehensive assessment of EBPs should always take a cross-setting, multi-informant approach to assist with understanding triggers for EBPs, clinical decision-making and care planning. Young autistic children displaying elevated EBPs at home, may not display this at school, and vice versa. Another key clinical implication is that an assessment of parenting stress should be conducted during care planning for autistic children, as recommended by the National Institute for Health and Care Excellence guidance for assessment of autism and intellectual disability and challenging behavior (National Institute for Health & Care Excellence, 2015, 2016). This is especially the case if discrepancies in parent and teacher reports, or observations of child EBPs are evident. An objective observation of the child in the home environment may aid clinician understanding of whether more parent-reported EBPs is because EBPs are primarily manifesting in the home environment. Given the differences in both the levels of EBPs and the modest agreement between parents and teachers, it may mean that different support for parents and teachers to help them manage child EBPs is required. It is important that discrepancies in informant reports are not dismissed as measurement error or informant bias. Clinicians should consider whether variations in informant report might reflect true situational variation in EBPs.

## Limitations and Future Research

There are several limitations to the current study. There was limited information on the characteristics of teachers and schools, so it was not possible to explore if other characteristics (e.g. teaching experience, workplace stress, number of adults in the classroom) were associated with reports of EBPs. Future research could consider how teachers’ confidence in supporting children with EBPs and their own levels of stress are associated with reports of EBPs. The observational measure was conducted in a third context and child BTC, whilst displayed primarily when interacting with their parent, was rated by a researcher. We did not conduct observations in either the home or school settings, so we are unable to tease apart the differences in contexts from the differences in informants. In addition, the study is correlational in nature so causality cannot be established. Future research using longitudinal designs should explore how reports of EBPs change over time and which factors are associated with discrepancies between informants both cross-sectionally and longitudinally. Finally, given that these data are drawn from a trial of a parent intervention for EBPs in autistic children it is possible the sample represents motivated families particularly concerned about EBPs, which may impact on generalizability.

## Conclusions

The current study contributes to the literature on reported EBPs among young autistic children. It uses a well-defined, representative sample of young autistic children to explore whether various child and informant characteristics are associated with EBPs and discrepancies between informants. The study highlights that the use of multiple informants and naturalistic observation is important for the assessment of EBPs in young autistic children to obtain a comprehensive understanding of the individual across contexts and support clinical decision making (Mash & Hunsley, 2005). Discrepancies between reports seem to be common and possible reasons for discrepancies should be explored; parenting stress and situational specificity may be particularly important. Assessment of EBPs could be supported by more objective measures, such as physiological response and independent observations of behavior, which could also be incorporated in evaluations of interventions designed to reduce EBPs in autistic children.

## Supplementary Information

Below is the link to the electronic supplementary material.

Supplementary file1 (DOCX 94 kb)

## Footnotes

1 We use ‘autism’ to refer to Autism Spectrum Disorder (ASD; DSM-5) and ‘autistic children’ as this is the preferred term of some of the autism community (Kenny et al., 2016).

2 BTC are typically defined as a behavior that puts the individual, those involved in their care or services at risk of harm or exclusion, due to their intensity, frequency or duration (National Institute for Health and Care Excellence, 2015; Royal College of Psychiatrists, British Psychological Society, & Royal College of Speech and Language Therapists, 2007). This term was chosen as it was preferred by our IAMHealth PPI panels.
