# Supplementary material for: Parent, Teacher and Observational Reports of Emotional and Behavioral Problems in Young Autistic Children
Source: J Autism Dev Disord. 2022 Jan 13;53(1):296–309. doi: 10.1007/s10803-021-05421-x (PMC9889526; doi:10.1007/s10803-021-05421-x)
Supplement: Supplementary file 1 — Supplementary file1 (DOCX 94 kb) [file 10803_2021_5421_MOESM1_ESM.docx]

| Supplementary Table 1. Correlations between the child and informant characteristics and parent- and teacher-reported child emotional and behavioral problems and researcher-rated observed behaviors that challenge. | | | | | | | | | | | | | | | | | | | | | | |
| --- | --- | --- | --- | --- | --- | --- | --- | --- | --- | --- | --- | --- | --- | --- | --- | --- | --- | --- | --- | --- | --- | --- |
|  | **Outcome** | | | | | | | | | | | | | | | | | | | | | |
|  | Irritability (ABC) | | | |  | Hyperactivity (ABC) | | | |  | Behavioral problems (ACB) | | | |  | Emotional problems (ACB) | | | |  | Observed BTC rate  (OSCA–ABP) | |
|  | Parent | | Teacher | |  | Parent | | Teacher | |  | Parent | | Teacher | |  | Parent | | Teacher | |  | Observed, researcher-rated | |
|  | *r* | *p* | *r* | *p* |  | *r* | *p* | *r* | *p* |  | *r* | *p* | *r* | *p* |  | *r* | *p* | *r* | *p* |  | *r* | *P* |
| Verbal language group^a^ (Minimally verbal vs. verbal) | -.00 | .988 | -.02 | .857 |  | -.04 | .722 | -.16 | .168 |  | .07 | .537 | -.17 | .142 |  | **.22** | **.048** | .15 | .191 |  | -**.48** | **<.001** |
| Autism severity (ADOS–2 CSS) | -.14 | .221 | .20 | .089 |  | -.08 | .452 | .14 | .245 |  | -.19 | .090 | .07 | .553 |  | -.05 | .670 | .15 | .201 |  | .15 | .178 |
| Observed BTC rate (OSCA–ABP) | .10 | .379 | .18 | .122 |  | .15 | .189 | .14 | .238 |  | .07 | .558 | **.23** | **.048** |  | -.12 | .271 | .07 | .530 |  | - | - |
| School placement (Specialist vs. mainstream)^b^ | **-** | **-** | .01 | .944 |  | **-** | **-** | -.06 | .618 |  | **-** | **-** | -.12 | .292 |  | **-** | **-** | .19 | .100 |  | **-** | **-** |
| Parenting stress (APSI)^c^ | **.61** | **<.001** | .02 | .882 |  | **.55** | **<.001** | .08 | .485 |  | **.57** | **<.001** | .16 | .179 |  | **.37** | **<.001** | .02 | .888 |  | .01 | .901 |
| Parental wellbeing (SWEMWBS) | **-.39** | **<.001** | .01 | .904 |  | **-.51** | **<.001** | .08 | .484 |  | **-.39** | **<.001** | -.07 | .554 |  | -.20 | .071 | .08 | .493 |  | .04 | .727 |
| Parent-reported irritability (ABC) | **-** | **-** | .19 | .096 |  | **.75** | **<.001** | .02 | .896 |  | **.81** | **<.001** | **.24** | **.034** |  | **.45** | **<.001** | .01 | .951 |  | - | - |
| Parent- reported hyperactivity (ABC) | **-** | **-** | **.28** | **.015** |  | **-** | **-** | **.34** | **.003** |  | **.78** | **<.001** | **.42** | **<.001** |  | **.38** | **<.001** | .10 | .411 |  | - | - |
| Parent- reported behavioral problems (ACB) | **-** | **-** | **.23** | **.043** |  | **-** | **-** | .14 | .212 |  | **-** | **-** | **0.31** | **.007** |  | **.57** | **<.001** | .07 | .547 |  | - | - |
| Parent- reported emotional problems (ACB) | **-** | **-** | .02 | .849 |  | **-** | **-** | .03 | .824 |  | **-** | **-** | .02 | .866 |  | **-** | **-** | .05 | .684 |  | - | - |
| *Note.* *N* = 82 for parent and observational measures. *N* = 76 for teacher reports. Significant correlations are in bold. ABC = Aberrant Behavior Checklist; ACB = Assessment of Concerning Behaviour; ADOS–2 = Autism Diagnostic Observation Schedule, second edition; APSI = Autism Parenting Stress Index; OSCA–ABP = Observation Schedule for Children with Autism–Anxiety, Behaviour and Parenting; SWEMWBS = Short Warwick-Edinburgh Mental Well-being Scale.  ^a^0 = Minimally verbal; 1 = Verbal.  ^b^School placement was used instead of verbal ability due to substantial overlap in group membership between these variables. 0 = Special schools; 1 = Mainstream (Mainstream schools + Special units in mainstream schools.  ^c^Parenting stress scores and parental wellbeing scores were moderately correlated (*r* = -.47, *p* < .001). | | | | | | | | | | | | | | | | | | | | | | |

Supplementary Table 2. Differences in parent-teacher scores and combined parent-teacher scores.

| **Measure** | Difference in parent-teacher scores (*M*, *SD*) | Parent-teacher combined scores (*M*, *SD*) |
| --- | --- | --- |
| Irritability (ABC) | 6.41 (12.79) | 12.36 (7.77) |
| Hyperactivity (ABC) | 6.69 (14.97) | 20.28 (10.60) |
| Behavioral problems (ACB) | 5.57 (13.20) | 19.47 (9.05) |
| Emotional problems (ACB) | 6.57 (15.22) | 17.94 (7.95) |
| *Note.* ABC=Aberrant Behavior Checklist; ACB=Assessment of Concerning Behavior | | |

|   *ρ* = .07, *p* = .528 |  |   *ρ* = .12, *p* = .295 |
| --- | --- | --- |
|   *ρ* = .09, *p* = .427 |  |   *ρ* = .29, *p* = .011 |
| Supplementary Figure 1. Bland-Altman plots showing average agreement between parent- and teacher-reported emotional and behavioral problems. For each plot, the difference in raw scores between parent and teacher reports was plotted on the y axis. The x axis represents the average of the parent and teacher raw scores. Within each plot, the average difference score (parent – teacher score) is represented by the solid black line and 95% CIs are represented by the dashed grey lines above and below the average score line. A positive difference score indicates that for that individual child, the parental report is higher than the teachers, and vice versa. The association between the difference score and the combined parent-teacher average score is represented by the dashed black line, along with the Spearman’s rho test and associated *p* value. | | |
